# Perceived Barriers to Blood Flow Restriction Training

**DOI:** 10.3389/fresc.2021.697082

**Published:** 2021-07-08

**Authors:** Nicholas Rolnick, Kyle Kimbrell, Mikhail Santos Cerqueira, Ben Weatherford, Christopher Brandner

**Affiliations:** ^1^The Human Performance Mechanic, Lehman College, New York, NY, United States; ^2^Owens Recovery Science, San Antonio, TX, United States; ^3^Neuromuscular Performance Analysis Laboratory, Department of Physical Therapy, Federal University of Rio Grande do Norte (UFRN), Natal, Brazil; ^4^Aspire Academy, Doha, Qatar

**Keywords:** BFR training, KAATSU, occlusion training, resistance training, rehabilitation, safety

## Abstract

Blood flow restriction (BFR) training is increasing in popularity in the fitness and rehabilitation settings due to its role in optimizing muscle mass and strength as well as cardiovascular capacity, function, and a host of other benefits. However, despite the interest in this area of research, there are likely some perceived barriers that practitioners must overcome to effectively implement this modality into practice. These barriers include determining BFR training pressures, access to appropriate BFR training technologies for relevant demographics based on the current evidence, a comprehensive and systematic approach to medical screening for safe practice and strategies to mitigate excessive perceptual demands of BFR training to foster long-term compliance. This manuscript attempts to discuss each of these barriers and provides evidence-based strategies and direction to guide clinical practice and future research.

## Introduction

Low-load blood flow restriction (BFR) training is an expanding area of research focus in both clinical (1) and performance (2) settings due to its unique potential benefits in comparison to similar exercise performed without BFR. BFR training involves the use of a compressive cuff that is applied to the proximal-most portion of the limb to partially restrict arterial inflow and occlude venous return (3) and has been shown to accelerate metabolic accumulation in both resistance (4) and aerobic (5) training. Commonly, BFR resistance training is performed using loads as low as 20% 1 RM (3, 6) whereas BFR aerobic training is usually performed at <50% VO_2_max or walking speeds of 4–6 km/h (3), although some recent research has successfully applied it post-exercise after bouts of high intensity aerobic exercise (7, 8). Longitudinal studies have shown BFR typically outperforms low-intensity training without BFR in various domains pertinent to rehabilitation and fitness practitioners including muscle hypertrophy and strength (9), cardiovascular capacity (10), time to exhaustion (11), functional task performance (12) and post-exercise hypoalgesic response (13). Recent systematic reviews have also shown that low-load BFR training promotes similar muscle hypertrophy (14) and strength (15) gains as moderate to heavy load strength training (≥60% 1 RM), highlighting the important role BFR training may play in rehabilitation settings (1) to optimize performance in sedentary and recreationally active participants. Other reviews have begun to hypothesize on the mechanisms underpinning the benefits of BFR aerobic and resistance training (16–20) including in highly trained individuals, expanding the potential utility of this modality to elite sport.

**Figure 1:**
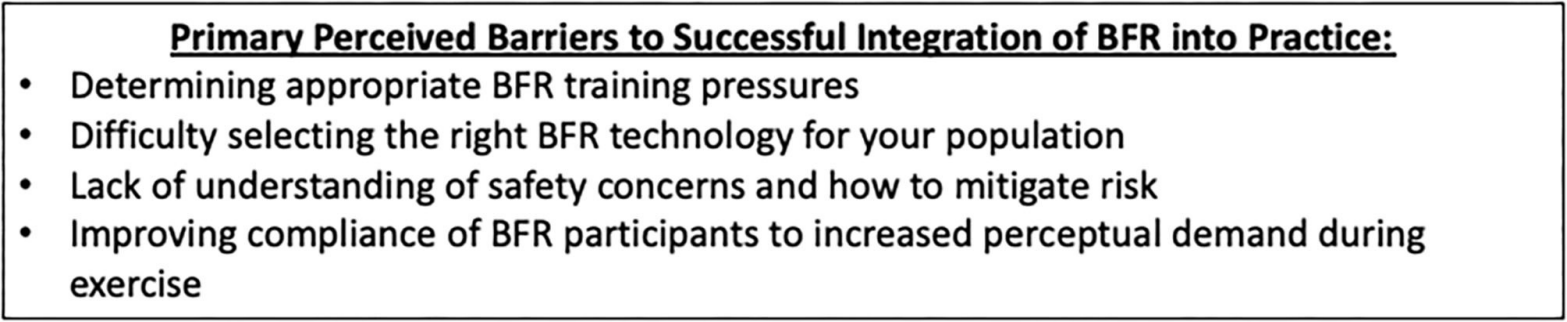
Primary barriers to BFR training integration. Primary barriers are those that are frequently encountered by BFR practitioners as they choose to integrate BFR into their respective plans of care (as determined through communications following BFR continuing education).

Despite the rapid increase in the BFR training literature supporting its use, practitioners seeking to use BFR may encounter a variety of perceived barriers to successfully incorporating it into their practices (Figure 1). These primary barriers include determining BFR training pressures, selecting an appropriate BFR training device, how to effectively perform a safety screening in a potential BFR training candidate and strategies to manage the elevated perceptual responses to BFR training to foster long-term compliance. The purpose of this manuscript is to discuss these common perceived BFR training barriers to provide valuable practice-based evidence recommendations and suggest strategies to facilitate a safe and effective BFR training environment to guide clinically relevant future research questions.

## Determining Barriers to Successful BFR Training

Barriers have been qualitatively determined through author consensus (NR, KK, BW) as clinician educators whose education companies have instructed and trained thousands of practitioners around the United States and abroad. BFR barriers were considered for inclusion based on the amount of post-training communications (via e-mail, social media, texts/calls) regarding the unforeseen challenges of successfully integrating BFR training into their practice.

### Blood Flow Restriction Pressure Assessment

One of the most significant barriers for practitioners incorporating BFR training in practice are the varied ways in which BFR can be applied. Different BFR applications range from the BFR device used (i.e., autoregulated pressures during application or standardized manual devices), the application form of BFR (i.e., continuously applied pressure throughout the exercise bout or intermittent application during exercise/rest periods only) and the use of various non-individualized BFR pressure prescriptions. At least two reviews (21, 22) have discussed the challenges of drawing conclusions using a variety of other applied pressure approaches including arbitrary pressure (i.e., 200 mmHg pressure for every participant) (23), pressures based off brachial systolic blood pressure (24), and pressures based on thigh circumference (25–27) as well as perceived tightness scale (i.e., “7/10” tightness, where “10” is maximum tightness) (28). While BFR training literature has utilized such approaches successfully in healthy participants, practitioners working in the clinical setting may benefit from more precise means of choosing and controlling pressure, especially in those with comorbidities.

Recently, Patterson et al. (3) proposed application guidelines recommending that the use of arterial occlusion pressure (or limb occlusion pressure, LOP) should be implemented in all research and practice settings to standardize the application of BFR training pressures. LOP is typically defined as the minimum applied pressure required to completely occlude both arterial inflow and venous return (3) for a given cuff (29) at a given time of day (30) in a particular body position (31). LOP can be established quickly and reliably using Doppler Ultrasound or built in pressure sensors of several commercially available devices (32, 33). Recent evidence also supports the use of pulse oximetry (34, 35) to determine complete blood occlusion in the upper limbs, although conflicting evidence exists for the lower limbs (35, 36). Pulse oximetry has also been shown to be impacted by race, limiting its generalizability outside of Caucasian ethnicities (37). While elaborations about the nuances of LOP is beyond the scope of this manuscript and have been discussed elsewhere (38), LOP allows a similar applied stimulus from client/patient to client/patient, standardizing the application and theoretically increasing safety.

After determining LOP, the next step is to decide what percentage of LOP will be used for exercise and training. Currently, the body of literature is limited with respect to information regarding specific participant responses utilizing LOP applications. Clarkson et al. (21) reported that 52 studies used LOP as a pressure application standard and only 16 of these were training studies. Further, their review highlighted a lack of sufficient rationale for applied cuff pressures, the level of application pressure as well as other important methodological considerations including cuff material. Typical recommendations for BFR pressures relative to LOP are equal to 40–80% (3). One study showed higher pressures (80% LOP vs. 40% LOP) positively impacted forearm vascular function similar to heavy load strength training, highlighting the potential for greater applied pressures to induce a preferential vascular adaptation despite similar levels of muscle growth (39). In addition, when lower loads are used (e.g., 20% 1RM), higher LOP pressures may be necessary to elicit muscle growth in non-failure exercise regimens (40). From a perceptual perspective, greater degrees of discomfort and/or exercise-induced muscle pain have been linked to higher applied pressures (41) and during the initial application period itself (42); therefore, while higher applied pressures may be optimal for a given goal, practitioners should be mindful of these perceptual demands as they may impact compliance with the intervention (see below section on perceptual demands). Thus, initiating care with physiologically suboptimal pressures may be a useful strategy to gradually expose clients/patients to the perceptual demands necessary to achieve adaptation with exercise.

While the research on LOP in BFR training is in its nascency, an individualized pressure approach as part of a multi-factorial decision-making process (see below section on medical screening) may be the best way to integrate BFR training safely into multiple settings and avoid unnecessarily exposing clients/patients to excessive exercise-related perceptual, cardiovascular, and hemodynamic responses. Excessive pressures can be avoided when prescribing based upon a percentage of LOP. Questions remain as to whether tissues other than muscle (e.g., bone, tendon, vascular) adapt in a similar fashion to a BFR intervention compared to traditional strength training. Variables such as the magnitude of pressure, and if the body position LOP is measured in influence longitudinal outcomes are of considerable interest. Nonetheless, there is a dire need for studies to implement a variety of BFR application pressures to discern these important methodological questions. Last, as more technologies are entering the marketplace, investigating the differences between acute and chronic application of these devices can help further shape clinical practice and establish safety profiles.

### BFR Training Technologies

There are currently multiple cuff technologies available to implement BFR in practice, which may make it difficult for practitioners to decide not only which are most effective to induce the desired training adaptations, but also potential safety features to minimize the risk of adverse events. BFR practitioners can overcome this barrier by understanding the different BFR technologies currently available. These can be broadly classified as tourniquets that can be defined as pneumatic (automatic autoregulated, automatic, or manual pneumatic cuffs) or non-pneumatic such as knee wraps/elastic bands (“practical BFR”).

Direct comparisons on neuromuscular, hemodynamics and perceptual responses between different restrictive approaches (i.e., pneumatic vs. practical BFR) are limited to acute studies (43–49). Interpretation of this small body of literature is challenging given that one (49) compared the resting blood flow responses between specialized elastic wraps to an automatic tourniquet of various pressures while the others investigated neuromuscular (45, 46), perceptual (44) and physiological (i.e., lactate and muscle swelling) (46) responses. It appears that practical BFR may generate similar acute changes in variables thought to induce positive musculoskeletal adaptations (i.e., muscle activation) (46, 48) while producing levels of perceptual demand that may be less than BFR training using pneumatic devices (44). However, practical BFR may not be suitable for clinical populations given it has the capacity to under- or overestimate applied pressures in the limbs by as much as 25% on a day-to-day basis (50). This raises some concerns about the reliability of this approach when working with individuals that may require more precise control of the BFR stimulus. While practical BFR has been shown to have efficacy in healthy people as well as athletes (2, 51), practitioners operating in a healthcare setting should look to other approaches that provide a more objective, reproducible stimulus.

To the authors' knowledge, only two studies have attempted to make direct comparisons of responses between BFR devices during acute BFR exercise (43, 47). Bordessa et al. (47) compared the acute electromyographic and perceptual responses and found that manual pneumatic cuffs (B-Strong™ Training System, 5 cm width at an unspecified but personalized pressure according to an algorithm created by B-Strong™) provide similar electromyographic activity as an automatic autoregulated cuff (Delfi Personalized Tourniquet System, 11.5 cm width at 80% LOP) but with lower levels of perceived exertion. In addition, Hughes et al. (43) compared the acute differences in cuff-limb interface pressure (set pressure vs. the pressure actually applied to the limb), hemodynamics and perceptual responses between a rapid inflation Hokanson device (13 cm width), a Delfi tourniquet system and a manual pneumatic cuff (8 cm width; Occlusion Cuff). During BFR exercise with 80% LOP, the autoregulated tourniquet device maintained similar pressures compared to the initial starting pressure, whilst the others did not. In addition, there were higher ratings of perceived pain during exercise and increased mean arterial pressures post-exercise with the rapid inflator device and the manual pneumatic cuff, but not the autoregulated tourniquet device.

Despite acute differences in cuff-limb interface pressures, a wide variety of BFR devices have shown favorable gains in muscle size and strength. A recent meta-analysis reported an increase of ~7% in muscle mass and ~14% increase in strength following 4–16 weeks of BFR training (14). On subgroup analysis, Lixandrão et al. (14) found that applied pressures, cuff widths and application of pressure prescription (individualized or arbitrary) did not influence muscular adaptations following training – supporting the use of multiple different devices to induce muscle mass and strength gains during BFR training. However, more research is needed to determine if all devices (automatic autoregulated, automatic, manual pneumatic cuffs, or practical wraps) result in similar long-term adaptations.

Practitioners' use of cuff technologies may ultimately rely on a stratified risk analysis based on the populations that they find themselves training or treating. Automatic autoregulated technologies may best be suited for post-operative and frail clients whose hemodynamic and cardiovascular systems may be more compromised as the evidence does show potentially exacerbated responses with ischemic exercise (52). Further, the ubiquitous adoption of surgical grade medical tourniquets during orthopedic surgeries have produced minimal complications (53) despite long durations (≥40 min) of supra-occlusive pressures. Application of BFR exercise pressures with similar technology adapted to the clinical setting may share a similar safety profile, reducing the potential for adverse tourniquet application sequelae (i.e., nerve injury, vascular damage etc.) during short bouts (5–20 min) of exercise with sub-occlusive pressures. Based on current evidence (14), manual pneumatic cuffs may best be suited for populations where potentially large fluctuations in hemodynamic/perceptual responses are not as much of a concern given appropriate BFR training exercise prescription. Practical BFR (i.e., use of knee wraps) is not strongly recommended in clinical practice despite its efficacy in the literature because of its lack of consistent reliability (50) and inability to accurately assess and program exercise at a percentage of LOP. We suggest that it is the practitioner's responsibility to uphold integrating BFR training with practices that align with research to minimize the risk of unnecessary adverse events. As performing BFR has some inherent risks [albeit low according to the epidemiological data (54–56)], use of BFR technology that can meet the bare minimum of what most research has deemed the standard of care (i.e., LOP) is important in ensuring the safest of BFR training practices.

### Safety Concerns

The available research coupled with the rapid expansion of BFR in clinical practice informs the overall safety of this intervention (3, 55, 57, 58), and thus developing a strategy for determining when to use or not use BFR is critical. Practitioners may understand the benefits of BFR training but given there have been reported safety concerns (53, 59), it's imperative they are able to quickly reason through those that are unique to BFR, as well as have a strategy for arriving at a sound clinical decision when presented with less common medical histories.

While scoring systems and algorithms may be helpful in ensuring that one has been thorough in a decision-making process, one barrier these possess in determining appropriateness is they may unwarrantedly increase perceived intervention risk in a medically complex population (53, 59). Clinical decision-making can be aided by pre-screening questions or questionnaires incorporated into new client/patient initial evaluation documentation, while other decisions hinge upon the subjective interview and physical examination. Due to the novelty of BFR training and the lack of empirically based guidelines for inclusion or exclusion, the execution and documentation of a thorough examination informed by the available literature related to safety is critical to justification of one's decision to use BFR.

Three primary areas of concern relating to BFR training identified in the literature are venous thromboembolism (VTE), potentially excessive hemodynamic/cardiovascular responses and muscle damage (3). Understanding what the available literature has demonstrated regarding these concerns is paramount to the safe use of BFR and will assist practitioners in the manipulation of variables such as load, pressure, effort, and volume to further the safety profile.

#### Venous Thromboembolism

Whether or not BFR exercise increases risk for VTE formation is likely the most thoroughly studied safety concern to date and a primary area of concern for individuals recovering from orthopedic surgery. In the first 6 weeks following orthopedic surgery, there is an estimated 100-fold increase in risk of VTE (60) secondary to the combination of “endothelial damage” and “stasis”: two of the three components that comprise Virchow's Triad. However, current evidence suggests that use of a tourniquet in surgery (“stasis”) does not seem to amplify this risk (61). Given the application of up to 120 min of full occlusion during orthopedic surgery, this prospective data should reduce concerns regarding risk of acquiring a VTE during or following the application of a brief (5–20 min), sub-occlusive pressure with BFR exercise. To date, no study has provided any evidence that BFR exercise amplifies markers associated with the coagulation system (62–64). For further reading on BFR exercise and potential VTE risk following orthopedic surgery, the reader is referred to Bond et al. (65).

#### Hemodynamics

As BFR exercise is more routinely used in clinical settings, practitioners need a working knowledge of how the intervention influences hemodynamics. The exercise pressor reflex (EPR) plays a strong role in the elevation of blood pressure and heart rate in response to exercise. The EPR was first detailed by Alam and Smirk in 1937 (66) through the use of a BFR exercise intervention and continues to be a key point of safety concern for BFR training as its adoption becomes more widespread, particularly in the clinical setting. That's because BFR training likely influences both the mechanical and metabolic arms of the EPR due to the compression of the limb via the cuff (mechanical) and the restriction of venous return stimulating the III-IV afferents (metabolic) (67). Populations with comorbidities such as hypertension (68), obesity (69) and diabetes (70) may exhibit altered EPR responses during exercise. Thus, concerns regarding the application of BFR during exercise in medically compromised clients/patients have been discussed (67, 71). Other groups have attempted to compare the hemodynamic response to BFR exercise to heavy load strength training to determine relative safety profile. The results are somewhat conflicting in design (i.e., arbitrary pressures vs. LOP), but indicate that BFR resistance exercise has the capacity to increase hemodynamic response to similar or even greater levels in healthy (72, 73) and hypertensive (74–76) individuals. A recent systematic review concluded that despite these elevations in hemodynamics, the responses appear to be within normal, tolerable limits – even for those with medical comorbidities (77). Nonetheless, despite researchers commonly using hemodynamics as an outcome measure, the responses to both BFR resistance and aerobic exercise have yet to be examined in a systematic way to comprehensively elucidate the various potential interactions of BFR applications. These include unilateral vs. bilateral, upper body vs. lower body, single joint vs. multi-joint exercise, automated vs. non-automated, higher vs. lower personalized pressures and of course, medically compromised vs. healthy participants. Mitigating excessive exercise-induced increases in hemodynamics likely can be partially attenuated by applying BFR intermittently (78, 79) as opposed to continuously as in standard BFR practice recommendations. For more reading on hemodynamics and the EPR, the reader is referred to these references (67, 71).

#### Muscle Damage/Rhabdomyolysis

It should be stated clearly that simply because a light load is used and may not cause a mechanical disruption of the myofiber, does not eliminate the risk for muscle damage with BFR training. This is supported by the fact that there are at least four cases of rhabdomyolysis associated with the intervention (80–83). Rhabdomyolysis is defined as the excessive release of creatine kinase and muscle myoglobin into the blood stream following excessive exercise-induced muscle damage (84) but not always (85). However, there is debate on the clinical importance of post-exercise elevations of creatine kinase and myoglobin absent of other clinical symptoms (i.e., myoglobinuria, malaise, weakness etc.) indicating rhabdomyolysis (84, 86).

While different cuff technologies (i.e., pneumatic vs. non-pneumatic) are thought to have varying abilities to mitigate safety risk during BFR training, these occurrences of rhabdomyolysis have been reported using a variety of cuffs and pressure prescriptions (80–83). It appears that unaccustomed BFR exercise, participant characteristics and/or the initial practitioner prescriptions are the greatest factors that influence negative major adverse events during BFR training, not necessarily the design of the cuff itself. Below we will provide some guidance regarding screening the client/patient to minimize risk, but a universal approach which might be deployed with traditional exercise of gradually progressing effort/intensity is likely best practice for promoting safe implementation. Tracking indirect markers of muscle damage such as delayed onset muscle soreness, range of motion loss, strength loss and edema should also be used to identify when to progress exercise more conservatively (3). Combined with sound clinical reasoning and integration principles (see below section), the risk of excessive muscle damage can likely be mitigated.

## Medical Screening for BFR Training

In this section, we propose a novel BFR training screening funnel and procedure to help overcome practitioner hesitancies to integrate this modality in practice. Practitioners wishing to incorporate BFR into a plan of care should be aware of the current consensus of the literature base and use a standardized assessment and screening protocol.

While screening and documentation should be performed prior to every session, the initial few exposures to BFR likely carry the greatest risk (86) for adverse events. Performing a thorough subjective examination and medical history that includes, but is not limited to, any history of cardiovascular disease, clots, clotting disorders, or rhabdomyolysis is pertinent and may substantially shape the clinical decision-making process. Kacin et al. (59) has previously developed a screening tool and others have written somewhat extensively regarding the safety of the intervention and developed risks and contraindication lists (3, 53). However, there has been no attempt to provide a thorough clinical reasoning procedure, and no tool has been validated to aid the practitioner's decision to use or not use BFR.

In addition to a subjective exam, the objective examination should include measurements of resting and exercise blood pressure and heart rate, general presentation of the client/patient and the limb(s) to be used, as well as any signs or symptoms of VTE.

**Figure 2:**
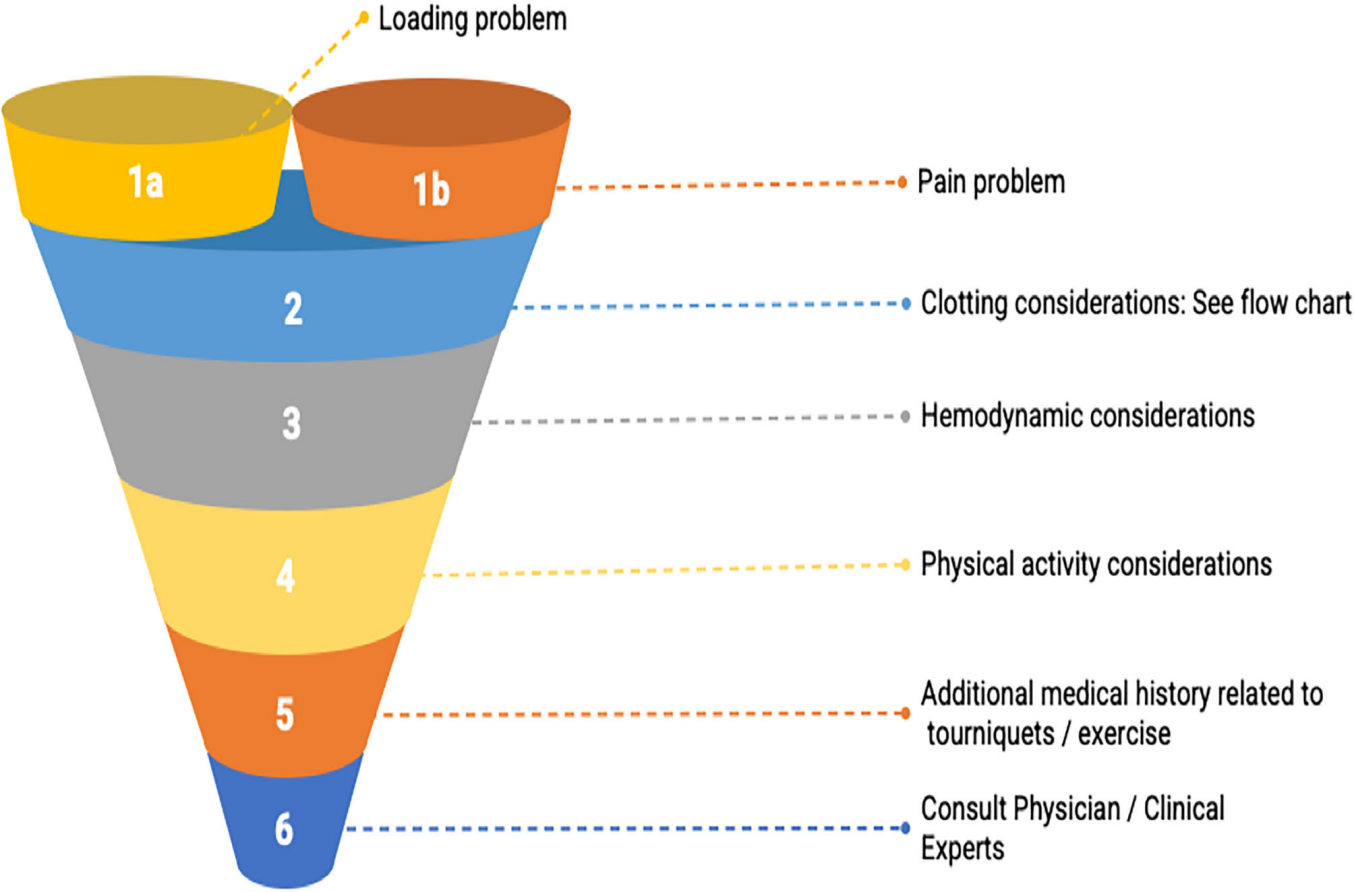
BFR decision making funnel. The goal is to guide practitioners' thought processes in an evidence-informed manner to consider relevant participant-related characteristics as they relate to the existing literature body so that a shared, informed decision to use or not use BFR can be reached. Note that the entry-point into the decision-making funnel involves determining whether the client/patient has a loading (i.e., unable to lift heavy weights) or a pain problem.

The decision to use BFR should not be diagnosis based. There is limited clinical data across all populations and thus practitioners should avoid claiming that BFR improves outcomes for any specific diagnosis. We have developed a clinical decision funnel for the inclusion or exclusion of use of BFR as a musculoskeletal rehabilitation tool (Figure 2). However, practitioners will inherently encounter medical histories and presentations for which it will be impossible to stratify risk if solely operating within the current body of literature. Therefore, it can be helpful to have a framework that allows a reasoned evidence-based decision. We've constructed our funnel to move from what we have good evidence for to presentations for which there may be no evidence whatsoever. The funnel presented below is best applied in the clinical orthopedic setting although other settings (i.e., cardiac rehabilitation and/or neurological) may also benefit as the limitations to exercise may be similar, but the body of research is sparse on conditions outside of orthopedics.

We hope our funnel builds upon others' work and encourage future trials that examine related topics in a systematic fashion to assist the practitioner in decision-making.

### Loading Problems

We propose a decision-making funnel that has two entry points. These points have been determined based upon the available literature and consensus agreement of the authors. Our first entry-point requires that the practitioner is navigating a presentation which substantially impacts the ability to load in accordance with ACSM guidelines (87). Rehabilitation practitioners routinely navigate numerous barriers that may make heavy resistance training, high-intensity interval training, or steady-state aerobic exercise difficult or impossible. Some of those barriers are perceived and have been the subject of position statements to address them. For example, APTA in their *Choosing Wisely* contribution states that physical therapists should avoid underdosing strength training exercises for older adults (88). Other barriers to loading are post-operative precautions where loads past a certain threshold may endanger the surgical site. To our knowledge, an additional barrier that is largely unaddressed thus far is imposed by those who have cardiovascular disease that may make previously mentioned exercise modes difficult or potentially unsafe. Finally, lesser prevalent but substantial barriers to loading exist like a client/patient's inexperience or fear with loading heavier or a lack of appropriate equipment in a clinical or home setting where care may be taking place.

Presently, there is robust evidence to support the use of BFR to provide an adaptation stimulus to muscle (i.e., hypertrophy) (9, 14, 89). Practitioners should take care to confirm the presence of a loading problem in the absence of specific directions from referring providers to limit load. Measuring force output for specific muscle groups via handheld dynamometry or a 5–10 RM test can help confirm a loading problem, as well as be used for exercise prescription and monitoring progress.

### Pain Problems

The second entry point to our funnel places emphasis on the goal of the BFR intervention within the context of research-based evidence on minimizing muscle/joint pain during and/or after exercise (42, 90–92). Tennent et al. (93) were the first to assess pain as an outcome associated with a BFR intervention. In a post-meniscectomy cohort, they demonstrated that the addition of BFR created a greater improvement in knee pain while achieving greater increases in strength and power relative to the same intervention without BFR. Other studies in the upper extremity in patients recovering from closed non-operative and operative distal radius fractures noted superior pain relief with BFR and better self-reported function than traditional care (94, 95). These results along with those from Hughes and Patterson (13) have fostered curiosity (96), and studies have begun to elucidate potential mechanisms as well as the influence of variables like applied pressure. Using BFR exercise for the specific purpose of reducing pain in a painful joint or limb in a therapeutic fashion or as a means of creating a heavy-loading window both seem reasonably defensible.

### Clotting Considerations

**Figure 3:**
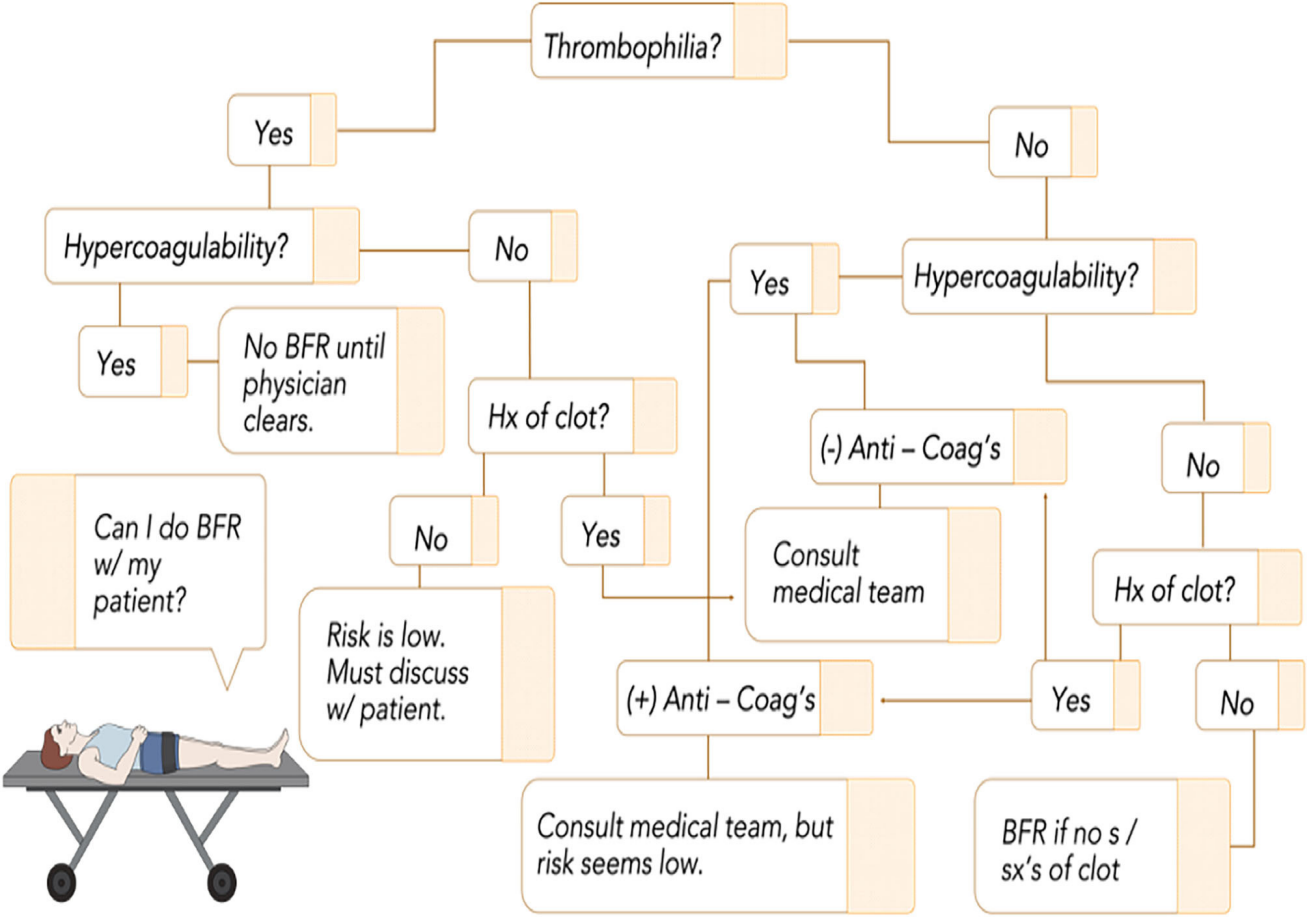
BFR flowchart as it relates to clotting risk. Given the low incidence of inherited thrombophilias, it is important to screen this initially as when combined with stasis or endothelial damage, it exponentially increases the risk for clot formation. From there, consideration can be given to individual circumstances to ensure that no major risk factor is inadvertently overlooked. The tree follows a yes/no format that leads to a recommendation that considers current best evidence. Refer to Bond et al. (65) for discussions on acquired hypercoagulability. Anti-Coag's, anticoagulant medication; Hx, history; s/sx's, signs and symptoms; BFR, blood flow restriction.

The second gradation in our funnel begins to address the overall safety as it pertains to comorbidities. VTE is a serious and potentially life-threatening condition common following orthopedic surgery. Whether or not BFR amplifies the coagulation system is a long-standing question that several studies have sought to elucidate (62, 64). Presently, no study has demonstrated amplification of direct markers of the coagulation system. For example, one study (93) showed that rehabilitation using BFR following meniscectomy did not produce differences in incidence of VTE compared to traditional physical therapy. Current clinical practice guidelines for those diagnosed with VTE suggest “strong” evidence in favor of activity and the use of intermittent pneumatic compression (97). Figure 3 displays a screening question and recommendations to the practitioner that can be used to help reason through this section of our funnel as, despite the preponderance of evidence on anti-clotting promotion with BFR training, there exists concern that BFR could lead to clot formation. Our decision tree begins by screening for any inherited thrombophilias. Practitioners should familiarize themselves at least with the more common thrombophilias like Factor V Leiden so they can perform this initial step seamlessly as part of their intake interview. Having a thrombophilia exponentially increases the likelihood of VTE following surgery due to the coupling of endothelial damage and stasis (“acquired” hypercoagulability); absent of specific direction from a referring specialist, this likely constitutes omission of BFR until guidance can be received or sufficient time post-op has passed. Those who do not have a thrombophilia have a much lower risk of VTE, but consideration must be given to a variety of presentations as risk can be amplified substantially, albeit temporarily, when using BFR in a rehab setting.

### Hemodynamics

Likely of the greatest concern given the known mechanical and metabolic components of the intervention is how BFR influences the exercise pressor reflex (EPR) (67, 71). As stated earlier, to the authors' knowledge, no one has approached answering this question in a systematic fashion. Several publications have gathered hemodynamic data and unlike the questions surrounding muscle damage, no adverse events (i.e., stroke or myocardial infarctions) have been reported - including in our clinical experience. There is even some indication that the intervention may be safe in those with controlled hypertension (54, 98, 99).

This section of the funnel relies in part on objective measurement. Practitioners should not implement BFR without screening blood pressure to ensure that their client/patient is safe for exercise. A number of groups (100–104) have recommendations for when to avoid resistance training or thresholds for cessation based upon intra-exercise hemodynamic responses in normotensive and hypertensive participants. Special consideration for further reading should be made to Sabbahi et al. (103) and Severin et al. (104) as these recommendations provide additional context and nuance to the discussion of pre- and peri-blood pressure responses with respect to age, gender and hypertensive status. The same recommendations should be adhered to with BFR exercise. Measuring LOP each visit and recording this information along with the duration of pressure applied to a limb or limbs is also pertinent, although LOP likely does not change significantly in most individuals on a day-to-day basis if exercising at a similar time of day (30). This is not only necessary for appropriate record-keeping but may also serve as a surrogate for blood pressure measurement after several exposures given the strong tie of LOP to systolic blood pressure.

### Physical Activity Considerations

As we near the exit of our funnel, it gets more difficult to provide strong recommendations based upon empirical data. Unaccustomed exercise is a risk factor for rhabdomyolysis regardless of the presence of medical comorbidities (84, 86). Risk of acquiring rhabdomyolysis following BFR exercise appears to be extremely rare based on the current state of the literature (3, 105) and is likely only elevated during the initial training sessions prior to acquiring the repeated bout effect (84).

In this gradation of the funnel, BFR practitioners should take into strong consideration the recent physical activity history of the client/patient. It's common for persons attending rehabilitation to be deconditioned for months or even completely unfamiliar with any sort of resistance exercise. As such, practitioners should screen recent physical activity history (with a special emphasis on resistance exercise) and extra care should be taken when progressing exercise effort and volume if the client/patient has been sedentary for 6 months or more. Consideration of the exercise history can significantly shape the initial weeks of BFR training integration while minimizing risk of adverse events (see below section).

### Medical History

Regardless of the known effects BFR exercise can have on muscle or the growing data that BFR is a mostly well-tolerated and safe intervention, clinical data is currently insufficient to state with strong degree of confidence that BFR enhances outcomes or is safe for a particular diagnosis. Practitioners are often presented with diagnoses they are unfamiliar with or immediately raise concerns when considering BFR. Some groups have published contraindication lists that can serve as quality references (53, 106), but even those appear to arbitrarily include diagnoses/conditions that have either no empirical support or due to the nature of the condition, offer little if any likelihood of empirical data ever being gathered to either support or refute. One such condition is pregnancy. Many list this as a contraindication to BFR training over the concern of the unknown and the relationship to gestational hypertension or the increased likelihood of acquiring a VTE (65). Yet women who are accustomed to traditional high-intensity exercise continue to do so with modifications throughout most of their pregnancy without complications (107). In fact, one case study reported use of KAATSU (a form of BFR) training during the third trimester was acutely well-tolerated by the exerciser and the fetus (108). Thus, BFR practitioners must consider all aspects of the condition, along with the available evidence as it relates to intense exercise and tourniquet use in surgery to arrive at a well-reasoned decision.

### Consult Physician/Clinician Experts

Since rehabilitation practitioners often manage clients/patients from referral sources that are also involved in the recovery process, it's recommended to consult the referring practitioner when reasoning through a safety concern pertaining to use of BFR. They may possess valuable information that makes the decision easier. The novelty of BFR lends itself to safety questions, and practitioners should always exercise caution and utilize interventions that maximize the client/patient's recovery and safety; that may or may not include BFR.

When to commence BFR post-operatively is an important question that requires the practitioner to make an informed decision that considers the wishes of referral partners and clients/patients. Presently, there is little evidence to make a strong recommendation for when to begin BFR following a surgical procedure of any kind. One study began a passive application of BFR at 3 days post-op (109), and most recently it has been suggested by Noyes et al. (110) that 3 weeks post-surgery is safe. The authors of this manuscript work closely with several physician groups who routinely commence BFR within a week of procedures like meniscectomy or ACL reconstruction and report no knowledge of any adverse events occurring from early integration. Particularly in the lower extremity, beginning meaningful exercise intervention is important to limit the rapid effects of disuse. There is a need for researchers to design clinical trials with a primary aim of discerning parameters for safe implementation of BFR post-operatively; this data would be very useful to the rehabilitation community at large. For BFR practitioners working with post-surgical clients/patients, close collaboration with physicians and other referral sources is encouraged to determine an appropriate starting date for BFR training.

Ultimately a decision to use BFR should be a shared process that places the highest priority upon the client/patient's goals and wishes. It's incumbent upon the practitioner to identify risk to present to the client/patient in an unbiased fashion, and when appropriate, seek counsel from referral sources and those who have more experience with the intervention.

We proposed this clinical decision-making funnel to address one of the largest perceived barriers to successful integration of BFR training in the rehabilitation setting. While this funnel is not empirically validated (i.e., studied for adequate screening of all potential variables that may influence BFR training), it helps guide the BFR practitioner through pertinent thought processes we feel are crucial to maximize safety. As the literature is sparse regarding a comprehensive BFR training screening process, this funnel and VTE flowchart could serve as the crux of the medical decision-making process when determining appropriateness for BFR training.

## Understanding the Importance of Exertion During BFR Training

Once the practitioner has decided that BFR may be a valuable intervention and an adequate medical screening and decision-making process has been undertaken to deem their client/patient is safe to perform BFR, understanding how perceptual demands are elevated with BFR training is important to increasing the long-term adherence of the intervention. This section will briefly focus on an overview of the science behind elevated perceptual demands (i.e., exertion) during exercise with and without BFR before introducing how BFR alters perceptual demands relative to low- and heavy load strength training. This section will also comment on initial strategies to reduce perceptual demands in those who may be clear to perform BFR but whose BFR training motivations may be attenuated if exercise intensity is too aggressive. BFR practitioners can overcome this barrier by understanding the role that high amounts of exertion play in enhancing musculoskeletal outcomes and being able to effectively program BFR training sessions to reduce excessive perceptual demands to foster long-term BFR training compliance.

Perception of effort/exertion can be defined as a feeling of work associated with voluntary actions like exercise and is distinctly separate from feelings of muscle pain and fatigue (111). Effort during exercise is qualified using validated scales such as the CR-10 scale (112), the Borg RPE scale (113) or the OMNI-RES (114) tool. Each of these tools attempt to anchor participants to rating the effort (rate of perceived exertion, RPE) required to complete the task to their subjective feelings of exertion. RPE during exercise likely arises from the processing of sensory signals related to the corollary discharge (a copy of the signal sent to the activated muscle) originating from centers in the brain controlling voluntary muscle recruitment (111). Importantly, corollary discharge (measured as movement related cortical potentials) increases when greater muscle force is required by activating more motor units (specifically higher threshold motor units containing more type II fibers) (115). Therefore, in non-fatiguing contractions, heavy strength training requires a larger corollary discharge compared to the same exercise performed with lower loads, producing higher RPE values (116) and electromyographic activity (117). During low load strength training without the accumulation of muscle fatigue, additional motor units containing type II fibers are minimally recruited (118) because total muscle force production is low, leading to a smaller corollary discharge and lower RPE. Regardless of load, as fatigue accumulates during strength training and movement speed involuntarily slows, corollary discharge (and subsequently RPE) increases to maintain similar muscle force output (115, 119, 120) leading to the recruitment of additional motor units. According to the force-velocity relationship, slow contraction velocities produce greater muscle force than faster moving contraction velocities, creating high mechanical tension – one of the primary mechanisms thought to induce muscle hypertrophy (121). Therefore, with low loads under fatiguing conditions, high RPE values can be a surrogate for a beneficial training stimulus.

However, in the clinical setting, achieving a hypertrophic stimulus with load compromised clients/patients may be difficult given the required amounts of repetitions likely needed to slow contraction velocity a sufficient magnitude. BFR training has been shown to reduce the repetitions needed to reach volitional fatigue (122) – in essence, lowering the threshold of repetitions needed to elicit a beneficial training stimulus. As muscle fatigue produces greater corollary discharge, BFR elicits higher RPE values compared to the same amount of repetitions performed in free-flow (123). In addition, the accumulation of metabolites from the restriction of venous return elevates muscle pain/discomfort to levels approaching or exceeding heavy strength training (42), heightening the perceptual experience. Despite the chronic training benefits of low-load BFR on muscle mass and strength, clients/patients may not tolerate the elevated perceptual demands of BFR training – especially during initial applications. This is particularly relevant in sedentary/post-surgical clients/patients whose tolerance or apprehensiveness to exercise-induced stress is compromised. Reducing the magnitude of perceptual demands could increase the likelihood of long-term compliance in a BFR training program (124).

Based on the above, perceptual responses such as RPE, discomfort, or pain likely constitute a barrier to using BFR exercise. Parameters such as applied pressure and exercise load can influence perceptual responses and can be modified to reduce perceptual demands, especially during initial training sessions to maximize tolerance and compliance. In fact, lower applied pressures (between 10 and 50% LOP) (125–128) and lighter loads (127) induces lower RPE during BFR training. Alternatively, use of intermittent BFR (i.e., where the cuff is deflated during the interset rest period) and a short familiarization period can be strategies to mitigate the barrier of perceptual responses. Studies have shown that intermittent BFR produces similar or lower RPE than both continuous BFR and non-BFR training (129, 130), and also heavy load training (78, 131). Regarding familiarization, it has been shown that RPE was reduced after repeated sessions of BFR (132, 133). Avoiding muscle failure is another way to mitigate RPE during BFR exercise. Lixandrão et al. (134) observed lower acute RPE when BFR exercise was not conducted to failure, whilst other studies (135, 136) showed similar gains in muscle mass and/or function after chronic training programs utilizing a non-failure approach with less RPE than during failure training. Last, wider cuffs are associated with higher RPE than narrow cuffs when the same arbitrary pressure is prescribed for all participants (137). Considering cuff width influences the occlusion pressure necessary to reduce blood flow to muscles (138), participants exercising with wider cuffs and same arbitrary pressures could experience a greater BFR stimulus, which could unnecessarily increase RPE response. Standardizing pressure application to a percentage of LOP may mitigate these excessive responses between cuff sizes, although more studies are needed to support or refute this claim.

Some evidence-based recommendations to minimize the perceptual responses as barriers to BFR training are: (i) use lower and individualized pressures; progression/adjustments in BFR pressure could be considered throughout the training; (ii) narrow cuffs should be preferred over wider cuffs when not personalizing pressures; (iii) intermittent BFR can mitigate discomfort, although its effectiveness is not yet clear; intermittent BFR could be considered in a familiarization period in those not tolerating continuous BFR; (iv) training until failure should be avoided, especially in the initial BFR training sessions as it likely does not provide superior benefits for muscle mass and strength; (v) familiarization periods should be considered whereby individuals perform a modified exercise protocol or under-dose by using loads < 20% 1 RM; (vi) communicate to the client/patient about the importance of high effort levels during BFR exercise with the long-term goal to transition these improvements toward optimizing function.

## Conclusion

BFR training is rapidly growing in the rehabilitation and fitness settings. Despite this growth, aspects such as understanding appropriate pressure application guidelines, the variety of BFR technologies, safe implementation in practice and the importance of perceived exertion in training to foster long-term compliance may be barriers to successful integration into the plan of care. This manuscript has attempted to discuss evidence-based and practice-based evidence solutions for the perceived primary barriers that currently exist in clinical practice from our experiences as clinician educators and researchers. Our goal is to help the reader be more informed on how to safely and effectively integrate this tool to optimize musculoskeletal outcomes. We also proposed a more comprehensive screening process including an evidence-informed funnel with a question tree to screen out individuals who may be at risk for a VTE. Future investigations could integrate this funnel into the screening process and look to validate it alongside other screening tools commonly used in practice.

**Figure 4:**
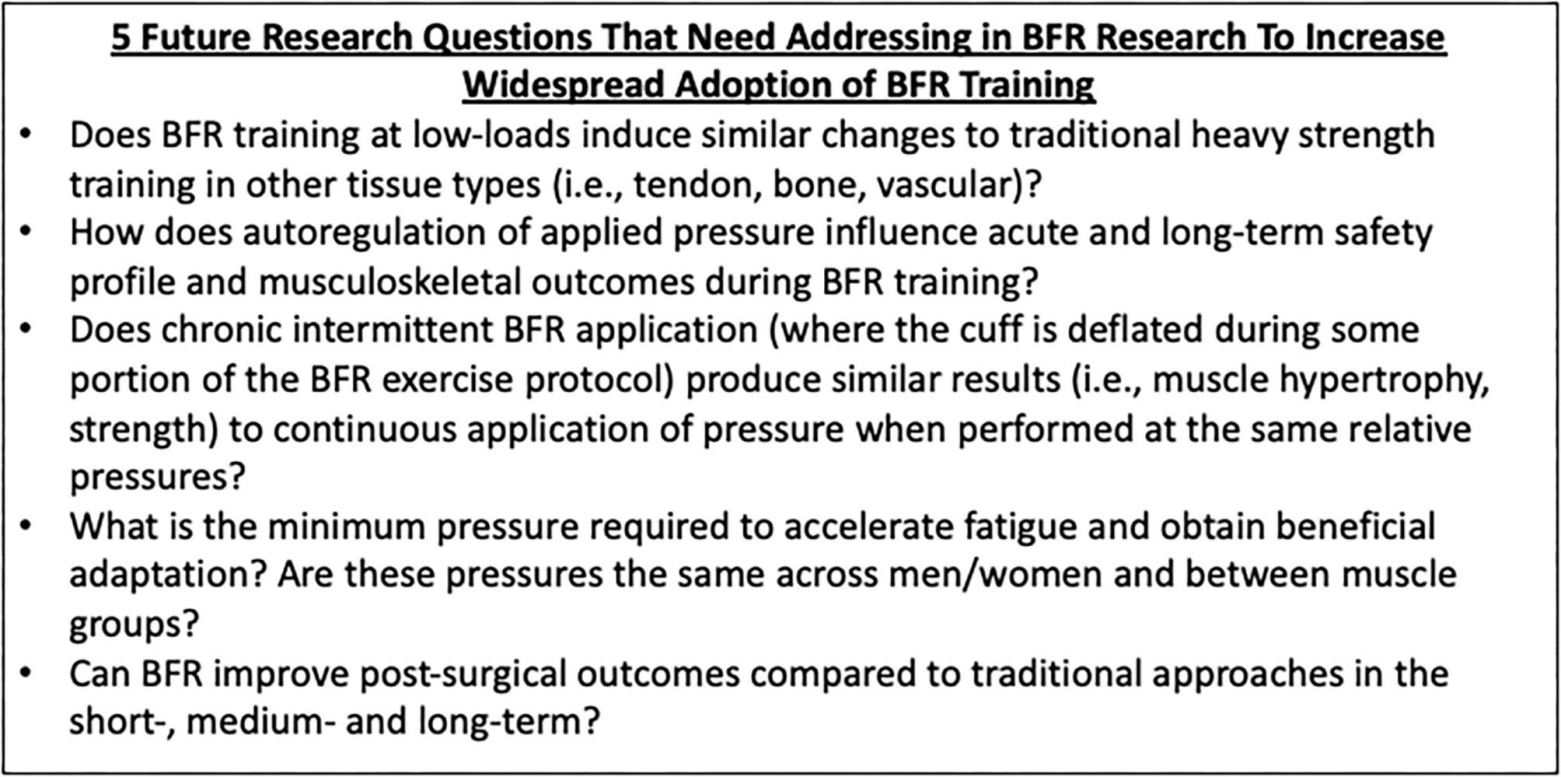
Five important BFR research questions. Five research questions for future investigation are presented here to highlight gaps in the current body of literature that may act as secondary barriers (not covered in this manuscript) to more widespread integration in practitioners across the healthcare/fitness continuum.

Last, our discussions in determining perceived barriers to successful BFR training generated clinically relevant research questions that have yet to be systematically addressed in the literature (Figure 4). If answered, these questions can help determine best practice of BFR training in rehabilitation and fitness settings and continue to grow this modality of training.

## Author Contributions

All authors contributed meaningfully to the writing and reading of this manuscript, involved in the design, and agreed to the statements made by the review.

## Conflict of Interest

NR is the founder of The BFR PROS and teaches BFR training workshops to fitness and rehabilitation practitioners using a variety of BFR training devices. KK and BW are clinical instructors for Owens Recovery Science, a BFR education company that also distributes the Delfi Personalized Tourniquet Device. The remaining authors declare that the research was conducted in the absence of any commercial or financial relationships that could be construed as a potential conflict of interest.
